# DEVELOPMENT OF OPEN EDUCATIONAL RESOURCES FOR AN AGING AND MENTAL HEALTH COURSE

**DOI:** 10.1093/geroni/igac059.2742

**Published:** 2022-12-20

**Authors:** Madison Ambrose, Jocelyn McGee, Sinai Wood, Chris Zakrzewski

**Affiliations:** Baylor University, Waco, Texas, United States; Baylor University, Waco, Texas, United States; Baylor University, Waco, Texas, United States; Baylor University, Waco, Texas, United States

## Abstract

The financial pressures associated with a gerontological education are significant given the rising costs associated with education. This fact is particularly relevant for economically vulnerable university students. One source of stress is the cost of textbooks and other required course materials. Indeed textbook prices have increased at a rate faster than other educational expenses. Additionally, the perspectives of diverse and underrepresented scholars are often not as readily available in some textbooks. In this presentation, our process of adapting an undergraduate Aging and Mental Health course to reduce cost and also showcase diverse perspectives is described in detail. The goals of this project were to: 1) adapt a preexisting course to include low- and no-cost content through the integration of open educational resources (OER); 2) provide the most up-to-date evidence based information for content and skills development in the area of aging and mental health; and 3) highlight the perspectives of diverse or traditionally underrepresented scholars in this field of study. We will also share the process of developing an interactive Pressbooks webbook from a strengths-based perspective wholistic perspective.

